# Evaluation of underreporting occupational accidents among workers who handle laboratory animals

**DOI:** 10.47626/1679-4435-2024-1275

**Published:** 2025-01-31

**Authors:** Flávia Soares Lessa, Isabella Brasil Succi, Gilberto Marcelo Sperandio da Silva

**Affiliations:** 1 Núcleo de Saúde do Trabalhador/Coordenação de Saúde do Trabalhador Fundação Oswaldo Cruz (Fiocruz), Rio de Janeiro, RJ, Brazil.; 2 Instituto Nacional de Infectologia Evandro Chagas, Fiocruz, Rio de Janeiro, RJ, Brazil.

**Keywords:** occupational accidents registry, underregistration, animals, laboratory, animal technicians, notificação de acidentes de trabalho, sub-registro, animais de laboratório, técnicos em manejo de animais

## Abstract

**Introduction:**

Occupational accidents pose a substantial health risk and represent a critical public health concern. While reporting occupational accidents is legally mandated, occupational accidents are significantly underreported, leading to institutional challenges and obstacles in planning and implementing preventive policies.

**Objectives:**

To evaluate how often workers who handle laboratory animals report their occupational accidents at the Instituto de Ciência e Tecnologia em Biomodelos/Fundação Oswaldo Cruz and to correlate this rate with their possible causes.

**Methods:**

This is an observational cohort study including workers who handle laboratory animals at the Instituto de Ciência e Tecnologia em Biomodelos/Fundação Oswaldo Cruz. Data were collected from questionnaires that these workers filled in and then compared to the accident records reported to the Núcleo de Saúde do Trabalhador (Brazil Workers’ Health Center) between 2014 and 2019. We used the Research Electronic Data Capture for data entry and Stata 14 for statistical analysis.

**Results:**

Occupational accidents were underreported by 44.8% of the workers who handle laboratory animals at the Instituto de Ciência e Tecnologia em Biomodelos/Fundação Oswaldo Cruz. The characteristics of those who mostly underreported were women (63.6%), of mixed-race (55.6%), 35-44 years old (53.3%), with a doctorate degree (80%), and permanently employed (50%). The most common types of occupational accident reported in the questionnaires were scratches (17.2%) and bites (13.8%), while the most common injuries recorded were cuts (26.7%) and trips/falls (17.8%). Reasons for non-notification were apparent minor severity (57.2%), unwillingness to report (14.3%), no specific protocol to treat (14.3%), excessive bureaucracy (7.1%), and high occurrence (7.1%).

**Conclusions:**

The underreporting rate of occupational accidents is 44.8% at the Instituto de Ciência e Tecnologia em Biomodelos/Fundação Oswaldo Cruz. Workers’ awareness of the importance and compulsory nature of reporting should be raised to reduce this rate.

## Abstract (pt)

**Introdução:**

Os acidentes de trabalho são o maior agravo à saúde dos trabalhadores, sendo um importante problema de saúde pública. A notificação dos acidentes é uma exigência legal, mas há uma significativa subnotificação, levando a desafios institucionais e a obstáculos no planejamento e na implementação de políticas preventivas.

**Objetivos:**

Avaliar a frequência da subnotificação de acidentes de trabalho entre profissionais que manuseiam animais de laboratório no Instituto de Ciência e Tecnologia em Biomodelos/ Fundação Oswaldo Cruz e correlacioná-la a suas possíveis causas.

**Métodos:**

Estudo observacional de coorte, envolvendo trabalhadores do Instituto de Ciência e Tecnologia em Biomodelos/Fundação Oswaldo Cruz. Os dados foram coletados a partir de questionários preenchidos por esses trabalhadores e comparados aos registros de acidentes notificados ao Núcleo de Saúde do Trabalhador, no período de 2014 a 2019. Foram utilizadas as ferramentas Research Eletronic Data Capture, para a entrada de dados, e Stata (versão 14), para a análise estatística.

**Resultados:**

A subnotificação de acidentes de trabalho no Instituto de Ciência e Tecnologia em Biomodelos/Fundação Oswaldo Cruz foi de 44,8%. As características dos trabalhadores com maior número de subnotificações foram gênero feminino (63,6%), cor parda (55,6%), faixa etária de 35-44 anos (53,3%), escolaridade de nível doutorado (80%) e vínculo de trabalho estatutário (50%). Os tipos de acidentes mais relatados nos questionários foram arranhaduras (17,2%) e mordeduras (13,8%), enquanto as lesões mais registradas nas comunicações de acidentes de trabalho foram cortes (26,7%) e tropeções/quedas (17,8%). As causas alegadas para a não notificação foram pouca gravidade aparente (57,2%), falta de vontade (14,3%), falta de protocolo específico (14,3%), burocracia excessiva (7,1%) e frequência elevada de ocorrência (7,1%).

**Conclusões:**

No Instituto de Ciência e Tecnologia em Biomodelos/Fundação Oswaldo Cruz, a taxa de subnotificação de acidentes de trabalho é de 44,8%. Para reduzi-la, é necessário sensibilizar os trabalhadores sobre a importância e a obrigatoriedade da notificação.

## INTRODUCTION

Occupational accidents pose a major burden on the health of Brazilian workers.^1,2^ Despite advances in research and technology in occupational health and safety in Brazil, the Ministério da Previdência Social (Ministry of Social Security) reports a high rate of occupational illnesses and accidents at alarming levels, leading to significant economic and social impact.^2^ According to Article 19 of Law No. 8,213/91,^3^ an occupational accident is

“[an accident] that occurs as a result of work performed on behalf of the company or as a result of work performed by the insured persons referred to in item VII of Article 11 of this Law, causing bodily injury or a functional disorder that causes death or permanent or temporary loss or reduction of capacity for work.”

Notification of occupational accidents consists of reporting to the competent authorities that an accident has occurred, so that federal agencies can monitor the statistics and take actions to prevent it. In addition, notification is essential to ensure that the injured worker is guaranteed their labor rights arising from the accident. Regarding notification, the veracity of data related to occupational accidents in Brazil is questioned,^4^ becoming an obstacle to planning and implementing prevention policies. When notification is not provided, a gap is created and strategic planning for the prevention of occupational accidents is hampered: that is called underreporting.^1^

Studies conducted with nursing professionals in Brazil suggest that some occupational accidents are considered “minor and routine hazards,” and are underreported 2.5-fold.^5^ More seriously, a study^6^ conducted jointly by the Ministério da Saúde (Ministry of Health), Secretaria de Segurança Pública do Estado de Tocantins (Tocantins Public Security Secretariat), Ministério do Trabalho (Ministry of Labor) and Secretaria da Previdência Social (Social Security Secretariat) from 2007 to 2015 in Palmas, Tocantins, Brazil, found that even in cases of occupational accidents with fatal outcomes, classified as serious and compulsorily notifiable, underreporting varies 29-73%, depending on the database accessed. This suggests that underreporting is more prevalent and more serious than official data reveal.^6^

Occupational accidents are underreported in several professional groups: environmental workers, hotel and hospital cleaners, carpenters, construction workers,^7^ and health professionals.^8,9^ The later are professionals more commonly found in the literature to underreport occupational accidents, especially those involving biological material. However, the literature is scarce about underreporting of occupational accidents for workers who handle laboratory animals, complicating the proposition and implementation of prevention policies aimed at this group.

It is worth noting that, in addition to biological risk, this group may be at increased occupational risk because they are possibly exposed to physical, chemical, ergonomic, organizational, and psychosocial risks.^10^

This study aims to assess how often animal workers who handle laboratory animals underreport occupational accidents and to correlate underreporting with their possible causes.

## METHODS

This is an observational, cohort and retrospective study conducted at the Instituto de Ciência e Tecnologia em Biomodelos da Fundação Oswaldo Cruz (ICTB/Fiocruz) and the Núcleo de Saúde do Trabalhador (Nust). ICTB/Fiocruz is a technical-scientific unit at Fiocruz, responsible for the production and supply of biomodels, including laboratory animals, blood, and blood products.^11^

Nust is the department responsible for dealing with occupational accidents at Fiocruz and their notification. We recruited 134 workers who handle laboratory animals at ICTB/Fiocruz (breeding, maintenance, or laboratory), through an invitation, to obtain convenience sampling. The Ethics in Research Commitee of the Instituto Nacional de Infectologia Evandro Chagas (CEP/INI) approved the study; the Certificate of Submission for Ethical Appraisal 29170820.0.0000.5262 was issued on March 31, 2020.

The inclusion criteria were: ICTB/Fiocruz worker who handle laboratory animals, of either sex, who had been in the job for more than 6 months. The exclusion criterion was: ICTB/Fiocruz workers who handle laboratory animals who were off work during data collection.

The study procedures were conducted from June 10, 2020 to November 24, 2020. In the first stage, participants completed a semi-structured, self-reporting questionnaire during their working hours, with prior authorization from ICTB. After signing the Informed Consent Form (ICF), sociodemographic data and occupational accident data were collected: age, sex, ethnicity, level of education, marital status, position held at ICTB/Fiocruz, length of service in the position, year of admission to ICTB/Fiocruz, employment relationship, weekly working hours, shifts, model(s) of animal(s) handled, and occupational accidents experienced. We also looked at the description of the accidents (type and intensity), whether they could have been avoided, and whether there had been any notification with a work accident report (CAT).

In the second stage, data on occupational accidents recorded by Nust from 2014 to 2019 that generated a CAT were collected, excluding accidents that occurred beyond this time. The investigators assessed non-notified accident reports, which were included in the questionnaires, and classified them as either occupational accidents or not, based on their characteristics, using them in the study to compare them with notified accidents.

This study only considered typical occupational accidents, excluding occupational diseases and work-related diseases, which are legally treated as occupational accidents under Article 20 of Law No. 8,213/91,^3^ as well as the cases described in Article 21 of that Law, which are also treated as occupational accidents. The occupational accidents reported in the questionnaires were compared to the data on occupational accidents registered with a CAT by Nust.

The main outcome assessed in this study was the underreporting of occupational accidents. To assess their rate, we used the information provided by the animal technicians on whether they had reported occupational accident, and the data recorded in the CATs. To assess the types of accidents, we compared the reports of the participants with the descriptions of the accidents reported to Nust. As for the causes of underreporting, the animal technicians were asked directly about them in the questionnaire should they have failed to report the accident.

Once the electronic form with the sociodemographic data had been filled in, a database was created with the information on the participants included in the survey, using the Research Electronic Data Capture (REDCap) data imputation software.^12^ This enabled the study population to be described, and the rate of occupational accidents in the participants to be analyzed, describing the accidents, whether they were reported or not, and the reasons for non-notification.

The data on occupational accidents reported by the animal technicians in the questionnaire were compared with the data on accidents obtained from the CATs at the ICTB/Fiocruz from 2014 to 2019. Stata 14 was used for statistical analysis. Fisher’s exact test was used to determine the correlation between the variables investigated (risk factors: sex, age, level of education, etc.) and the primary outcome (underreporting). The tests used a 95% confidence interval. Therefore, results with an associated p-value of less than 5% (0.05) were considered significant.

## RESULTS

The sample included 53 volunteers from ICTB/ Fiocruz, representing 39.6% of the study population (134 animal technicians). Of these, 33 (62.3%) reported having had occupational accidents; however, only 19 (57.6%) reported them. They were mainly man (35; 66%) and white (25; 47.2%). As for their age group, 23 (43.4%) were 35-44 years old, and 28 (52.8%) were outsourced workers (Table 1). Additional sociodemographic and functional data on the workers who had occupational accidents and those who reported them are shown in Table 1.

**Table 1:** Sociodemographic and work-related characteristics of animal technicians at ICTB/Fiocruz who reported occupational accidents

Sociodemographic and work-related characteristics	n	%	n	%	n	%
Sex						
Male	35	66.0	22	66.7	15	78.9
Female	18	34.0	11	33.3	4	21.1
Skin color/ethnicity						
White	25	4 7.2	16	48.5	9	4 7.4
Black	13	24.5	7	21.2	6	31.6
Mixed-race	13	24.5	9	2 7.3	4	21.1
Yellow	2	3.8	1	3.0	-	-
Age group (years)						
Up to 24	2	3.8	2	6.1	2	10.5
25-34	11	20.8	6	18.2	4	21.1
35-44	23	43.4	15	45.5	7	36.8
45+	17	3 2 .1	10	30.3	6	31.6
Level of education						
High school	24	45.3	13	39.4	8	4 2 .1
College	11	20.8	7	21.2	6	31.6
Specialization	1	1.9	1	3.0	1	5.3
Master’s degree	12	22.6	7	21.2	3	15.8
Doctorate	5	9.4	5	15.2	1	5.3
Length of service (years)						
Up to 4	11	20.8	6	18.2	3	15.8
5-9	18	34.0	11	33.3	9	4 7.4
10-14	8	15.1	6	18.2	1	5.3
15+	16	30.2	10	30.3	6	31.6
Type of employment						
Scholar	5	9.4	1	3.0	-	-
Employee	20	3 7.7	16	48.5	8	42 .1
Outsourced employee	28	52.8	16	48.5	11	5 7.9

ICTB/Fiocruz = Instituto de Ciência e Tecnologia em Biomodelos da Fundação Oswaldo Cruz.

Of the 53 interviewees, 16 (30.2%) handled nonhuman primates. Additional data on the other types of animals is shown in Table 2.

**Table 2:** Types of animals the workers handled at ICTB/ Fiocruz (n = 53)

Animals workers handled	n (%)
Non-human primates	16 (30.2)
Mice/rats	12 (22.6)
Rabbits/guinea pigs	11 (20.7)
Rabbits/guinea pigs + mice/rats	9 (17.0)
Sheep/horses + rabbits/guinea pigs	3 (5.7)
Sheep/horses	2 (3.8)

ICTB/Fiocruz = Instituto de Ciência e Tecnologia em Biomodelos da Fundação Oswaldo Cruz.

Descriptions of the accidents were obtained from the participants. The 33 workers who handle laboratory animals who claimed they had occupational accidents reported a total of 49 accidents, all of which were classified as occupational accidents. Of these 49 accidents, 20 (40.8%) were excluded because they occurred beyond the study period. This resulted in a total of 29 (59.2%) accidents for analysis, as shown in Table 3.

**Table 3:** Total occupational accidents reported in the questionnaires by workers who handle laboratory animals at ICTB/Fiocruz, registered at Nust/CST (with a CAT) and accidents notified to Nust from 2014 to 2019, included for analysis

Type of accident	Total accidents reported in the questionnaires n (%)	Accidents reported to Nust/CST with a CAT n (%)	Accidents reported to Nust/CST and included for analysis n (%)
Bite	10 (20.4)	3 (6.7)	4 (13.8)
Scratch	8 (16.3)	1 (2.2)	5 (17.2)
Burn	4 (8.2)	3 (6.7)	2 (6.9)
Sharps with biological material	4 (8.2)	2 (4.4)	3 (10.3)
Cuts with no contact with biological material	3 (6.1)	12 (26.7)	1 (3.4)
Contact of biological material with mucous membranes	3 (6.1)	2 (4.4)	2 (6.9)
Crushing of fingers	3 (6.1)	3 (6.7)	3 (10.3)
Cuts with contact with biological material	2 (4.1)	3 (6.7)	1 (3.4)
Trips/falls	2 (4.1)	8 (17.8)	2 (6.9)
Fracture	2 (4.1)	1 (2.2)	-
Mild head trauma	2 (4.1)	1 (2.2)	1 (3.4)
Horse headbutts	2 (4.1)	-	2 (6.9)
Corneal injury	1 (2.0)	1 (2.2)	-
Severe muscle contracture	1 (2.0)	1 (2.2)	1 (3.4)
Acute allergic reaction	1 (2.0)	1 (2.2)	1 (3.4)
Halter restraint*	1 (2.0)	1 (2.2)	1 (3.4)
Allergy	-	2 (4.4)	-
Total	49 (100.0)	45 (100.0)	29 (100.0)

Nust = Núcleo de Saúde do Trabalhador; CST = Coordenação de Saúde do Trabalhador; ICTB/Fiocruz = Instituto de Ciência e Tecnologia em Biomodelos da Fundação Oswaldo Cruz; CAT = work accident report.

* The animal technician got caught in the halter during the procedure.

Of the 29 accidents analyzed, 9 (31%) were reported by workers who handle laboratory animals who did not report to Nust. The other 20 (69%) accidents were reported to Nust. It is worth noting that even these workers did not report all their accidents. Thus, of the 20 (69%) accidents mentioned, only 18 (62.1%) were reported, according to their information.

Therefore, the underreporting rate of occupational accidents in this study was 37.9%, based solely on the information provided by the workers. In addition, we obtained data relating to 45 occupational accidents with a CAT at ICTB/Fiocruz which were reported to Nust during the period shown (Table 3). This data were compared to the 29 accidents reported by the workers who participated in this study.

As reported by the workers who handle laboratory animals, of the 29 accidents mentioned in the questionnaires, 18 (62.1%) were officially reported to Nust. Of the accidents that the participants in the study mentioned, 16 (55.2%) were identified in the accident records notified to Nust, as shown in Table 4.

**Table 4:** Comparison between the accidents reported by workers who handle laboratory animals at ICTB/Fiocruz in the questionnaires and the accidents both reported in the study and reported with a CAT to Nust, from 2014 to 2019

Type of accident	Reported in the study n (%)	Reported in the study with a CAT n (%)
Scratch	5 (17.2)	-
Bite	4 (13.8)	2 (12.5)
Sharps with biological material	3 (10.3)	2 (12.5)
Finger crush	3 (10.3)	2 (12.5)
Trips/falls	2 (6.9)	1 (6.3)
Burns	2 (6.9)	1 (6.3)
Contact of biological material with mucous membranes	2 (6.9)	2 (12.5)
Horse headbutts	2 (6.9)	1 (6.3)
Cuts with contact with biological material	1 (3.4)	2 (12.5)
Cuts with no contact with biological material	1 (3.4)	1 (6.3)
Fracture	-	-
Halter restraint*	1 (3.4)	-
Mild head trauma	1 (3.4)	1 (6.3)
Severe muscle contracture	1 (3.4)	-
Acute allergic reaction	1 (3.4)	1 (6.3)
Chemical agent splash	-	-
Allergy	-	-
Total	29 (100.0)	16 (100.0)

ICTB/Fiocruz = Instituto de Ciência e Tecnologia em Biomodelos da Fundação Oswaldo Cruz; CAT = work accident report; Nust = Núcleo de Saúde do Trabalhador.

* The animal technicians got caught in the halter during the procedure.

**Figure 1:**
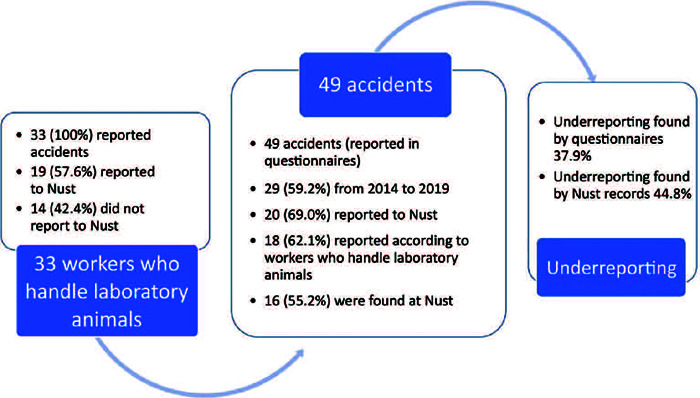
Details of how many workers who handle laboratory animals reported accidents or not. Number of accidents reported to Nust or not, and within the study. Details of how many accidents were reported according to information provided by the workers and how many were reported to Nust. Underreporting of accidents was measured in two different ways: firstly, based on the questionnaires and, secondly, based on Nust records.

When comparing the data provided by the questionnaires filled in by the workers who handle laboratory animals with the data from CATs, 13 (44.8%) of the 29 accidents were not reported. Therefore, based on the records, the underreporting rate in this group reached 44.8%, as shown in Figure 1.

When asked why they did not report to Nust, workers who handle laboratory animals said that the accident was not very serious (57.2%). The other reasons are shown in Table 5.

**Table 5:** Reasons ICTB/Fiocruz workers who handle laboratory animals claimed for not reporting occupational accidents to Nust and frequency (n = 14)

Reason for not reporting to Nust	n (%)
Minor accident	8 (57.2)
Unwillingness to report the accident	2 (14.3)
No protocol to treat the accident	2 (14.3)
Excessive bureaucracy	1 (7.1)
Frequent occurrence of accidents	1 (7.1)

ICTB/Fiocruz = Instituto de Ciência e Tecnologia em Biomodelos da Fundação Osvaldo Cruz; Nust = Núcleo de Saúde do Trabalhador.

## DISCUSSION

The perception of the workers who handle laboratory animals that an occupational accident represents a potential risk is directly linked to notification. Therefore, when these workers understand that occupational accidents pose an associated risk, they will be more inclined to report them.^13^ As underreporting of occupational accidents is a worldwide problem that affects many professions^7^ and prevents prevention policies from being proposed and implemented, the analysis of occupational accidents conducted in this study is useful for assessing underreporting and its causes, and for proposing the implementation of appropriate policies to meet the needs of workers who handle laboratory animals.

In this study, more than half of the participants had higher education or above. However, even among qualified professionals, occupational accidents occurred. Mid-level professionals had the highest rate of accidents, which was also observed in a study at a university hospital,^14^ which found that licensed practical nurses with no higher education accounted for approximately 80% of all sharps accidents.

That study^14^ also found that the workers who had the highest proportion of occupational accidents were the youngest, with length of service of up to 5 years. Occupational accidents occur more frequently at the extremes: inexperienced, poorly trained, and insecure workers^15^ and those who are confident enough to neglect safety protocols, with more than 15 years in the profession.^14^

Both the type of animal handled, and the position held by the animal technician influence the type of accident most frequently suffered.^16^ Veterinary technicians are known to suffer the most bites; veterinary residents, sharps injuries; and veterinarians, mucous membrane exposure to biological material.^17^ In this study, bites were the second most common accident among workers who handle laboratory animals. The animals which were handled by most of the workers in the study (in an isolated group) were non-human primates. Scratches are the accident that workers who handle laboratory animals most frequently reported in this study; they are also common among workers who handle smaller animals, such as rodents, and are generally reported together with bites.^18^

The rate of non-notification to Nust was 42.4%. This figure was obtained by evaluating the 33 (62.3%) ICTB/Fiocruz workers who handle laboratory animals who reported accidents, of whom 14 did not report them to Nust. However, each worker may have suffered more than one accident and, in this case, notified one and not the others. This phenomenon is consistent with the findings of a French study,^19^ which indicated that the more accidents a worker suffers, the less inclined they are to report them.

Accident underreporting should therefore be assessed using the number of accidents, not the number of workers injured. Thus, the underreporting rate was 37.9%, according to the workers’ reports in this study. However, when comparing these results (information provided by workers) with the notifications reported to Nust, the underreporting rate rose to 44.8%. The underreporting rates found in the literature vary, for example, from 45% among health workers in a Portuguese hospital^20^ to 70.2% among doctors in a French hospital.^19^

There is a difference between the type of accident notified to Nust and that reported by the group participating in the study. Most unreported accidents were found to be less serious and more frequent. Similarly to what was recorded in a review study on nurses, workers do not report accidents because they consider them to be minor and routine.^21^

Scratches and bites, which are typical accidents for workers who handle laboratory animals, were more frequent in the study group, while cuts and trips/falls were more frequently reported to Nust. This may be because cuts generally require medical assessment and/or suturing, as do trips and falls, which are also considered more serious and require medical care and/ or dressing.

Scratches and bites, on the other hand, although potentially serious due to the risk of contact/ contamination with biological material, and the risk of zoonoses and other diseases, are not perceived by workers as serious accidents, either due to their high rate or superficiality. Thus, when they do occur, they are treated on site and, if there is no need for suturing, there may be no demand for medical care. Consequently, the accident is not reported.

Underreporting of occupational accidents was more common in people with the following demographic characteristics: women of mixed race, between 35 and 44 years old, with a doctorate, 10-14 years of employment. Although accidents occurred proportionally between men and women, women reported less. The reason for this is that women already feel overloaded in their daily lives and are unwilling to add this task to the many they already must perform^22^; moreover, the notification process is cumbersome.^7^

This study found that individuals of mixed race were the ones who most often failed to report their occupational accidents. However, it was not possible to find data related to ethnicity in the literature that would allow to compare the underreporting rate of occupational accidents. Professionals who reported their accidents the least were those with a higher level of education, especially a doctorate. A similar patern was observed in a study of health professionals in Portuguese hospitals,^20^ in which physicians were the least likely to report accidents.

It should be noted that all the participants in this study with a doctorate reported having suffered an occupational accident, a finding that has already been highlighted. We can also add that occupational accidents may not have been reported because workers perceived them either as a lack of skill or as their own responsibility, failing to report them to avoid embarrassment.^23^

The main reasons given for not reporting occupational accidents to Nust include the apparent absence of severity and the unwillingness of the injured person to report to Nust. These reasons also appear in the literature, as mentioned in the study by Yang et al.,^23^ which investigated surgical residents in the United States. In that study, 21.6% of the participants claimed not reporting accidents due to no apparent severity, while 52.3% atributed the omission to unwillingness. In addition, 25.5% mentioned the negative association with people who have accidents with sharps, 7.5% reported pressure not to report, and 6.8% said they would not want to know the result of the test. However, participants reported that the most frequent cause (80.3%) was the time spent to notify, a significant factor that makes it difficult to comply with this procedure.^23^

Workers who handle laboratory animals at the ICTB/Fiocruz did not mention pressure not to notify or negative associations with people who have accidents with sharps, which may be because there are professionals at the ICTB who provide guidance to workers in the event of an accident.

In addition to these causes, another reason cited for non-notification was not having a protocol for dealing with certain types of accident. This was the case, for example, with accidents involving non-human primates that occurred when there was no protocol at NUST for such situations. Currently, there is a protocol for accidents involving biological material and nonhuman primates,^24^ and this reason for not reporting to Nust no longer applies. Unwillingness and insufficient time to report occupational accidents may mean that the notification process, even if not performed by the worker is laborious and time-consuming. In a North American study on the underreporting of accidents with sharps in medical residents,^23^ the main reasons they cited were “lack of time” and “too much bureaucracy.” Therefore, making this process easier and less bureaucratic could be an alternative to encourage the notification of occupational accidents.

Another way is to spread awareness about the legal obligation to report occupational accidents. This can also be an important strategy, since awareness of the laws tends to increase the likelihood of compliance.^25^

## CONCLUSIONS

This study showed that 44.8% of the workers who handle laboratory animals at ICTB/Fiocruz underreport occupational accidents. The factors most related to underreporting were the apparent minor severity (57.2%), no protocol for dealing with a particular accident (14.3%), unwillingness to report (14.3%), high rate of accidents (7.1%), which were also considered not severe, and excessive bureaucracy (7.1%). The most frequently reported types of occupational accident in the study questionnaires were scratches (17.2%) and bites (13.8%). The most frequent accidents reported to Nust were cuts (26.7%) and trips/falls (17.8%). The main actions to reduce underreporting rate are raising awareness and instructing workers that even apparently minor and unimportant injuries should be reported, speeding up the care procedure, emphasizing the legal obligation to report occupational accidents, and changing workers’ perceptions of occupational accidents.
